# In regards to “Feasibility study of in‐house second‐channel calibration of dual‐channel electrometers using a battery‐powered current source”

**DOI:** 10.1002/acm2.70617

**Published:** 2026-05-24

**Authors:** Shimizu Morihito

**Affiliations:** ^1^ National Metrology Institute of Japan (NMIJ) National Institute of Advanced Industrial Science and Technology (AIST) Tsukuba Ibaraki Japan

1

Dear Editor,

I would like to comment on the paper entitled “Feasibility study of in‐house second‐channel calibration of dual‐channel electrometers using a battery‐powered current source.^1^”

The Japanese Society of Medical Physics (JSMP) recommends that users periodically inspect electrometers (i.e., current and charge measurement devices) used as radiotherapy dosimeters. Although inspection by the electrometer manufacturer is generally preferred, the JSMP guidelines also allow users to perform inspections based on the output current and charge from the ionization chamber during radiation beam irradiation.^2^

In this context, Tsuno et al. proposed a method that combines a dry‐cell battery‐powered current source with a timer switch to generate both current and charge sources, with the aim of simplifying the inspection process. Following a patent^3^ in Japan for the charge source and the associated electrometer inspection system based on the JSMP guidelines, the authors have reported this approach in three publications: two in Japanese^4^, ^5^ and one in the present study.^1^

A charge measurement device is typically calibrated using a charge source composed of a constant‐current source and a timer switch. However, as illustrated in Figure 1a, the configuration described by Tsuno et al. as a current source consists of a dry‐cell battery connected in series with a resistor.^3^ From a circuit perspective, this arrangement is more appropriately characterized as a voltage source with high output impedance. Under such a configuration, the ability to generate a truly constant current or a well‐defined quantity of charge may be limited.

**FIGURE 1:**
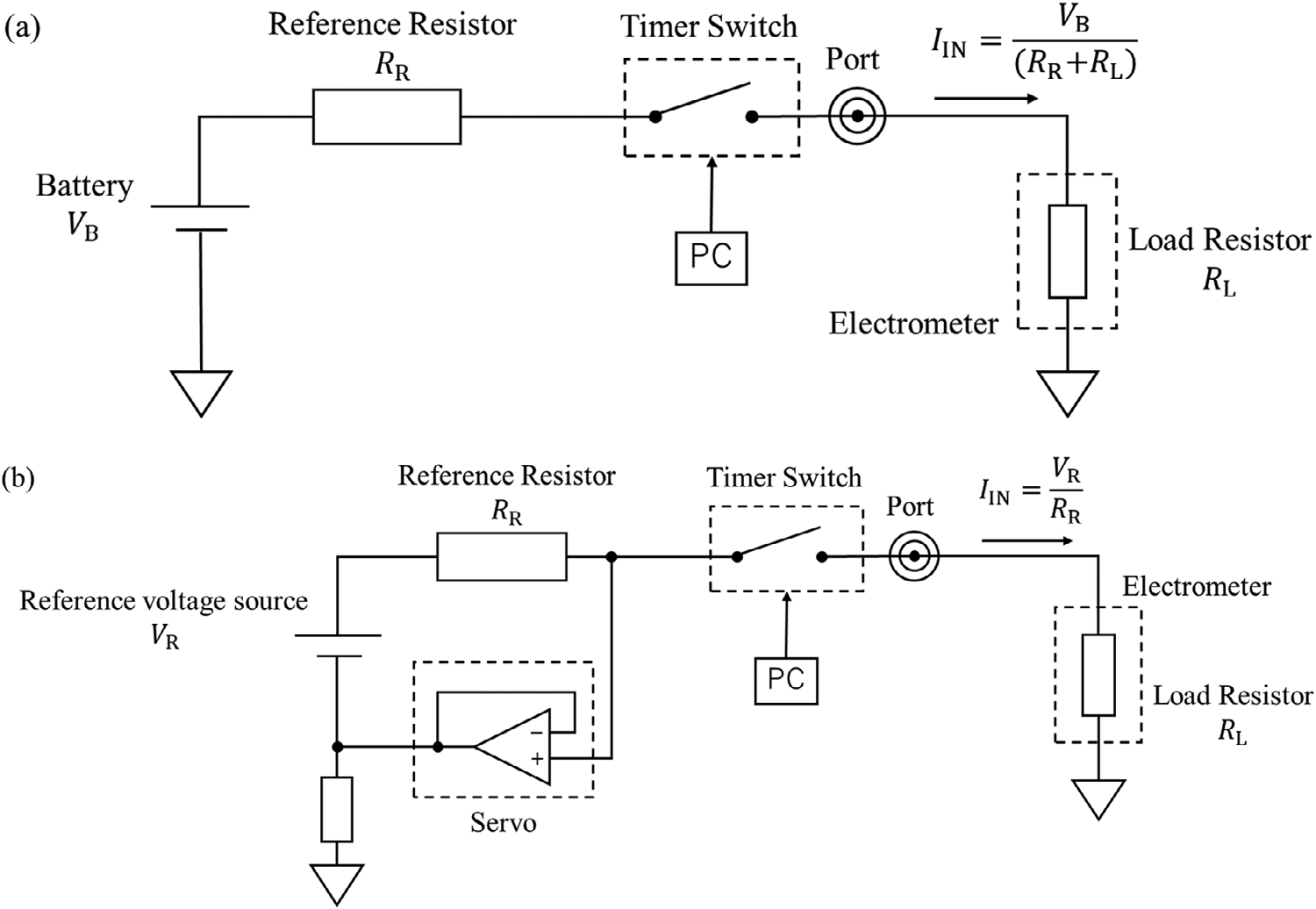
Schematic diagram of (a) the proposed charge source for electrometer calibration as proposed by Tsuno et al., and (b) the charge source used in standard laboratories. The illustration of the proposed charge source was based on the published patent information.^3^ The proposed charge source combines a reference resistor, dry cell battery, and timer switch. The current input from the proposed charge source to the electrical instrument under calibration depends on the electrometer's load resistance. The charge source used in standard laboratories has a servo circuit that maintains constant input current and suppresses excessive current generation, enabling the safe and accurate calibration of electrometers.

Tsuno et al. reported that identical readings were obtained from the reference electrometer circuit and the electrometer under test. However, the output current of the proposed current source depends on the internal reference resistance of the source and load resistance of the connected electrometer circuit. Hence, the current delivered may vary depending on the specific electrometer configuration.

Furthermore, as noted by Tsuno et al., differences in the value of the protective resistor within the electrometer can influence the input current. In addition, the input current may be affected by the open‐loop gain ($A_{v})$ of the electrometer's internal negative‐feedback circuit; lower gain values can lead to greater deviations. This effect may exceed 0.1% for low‐gain electrometers ($A_{v}$ < 1000), such as vibrating‐reed electrometers, and therefore may not be negligible. In this context, equivalence of displayed readings alone may not be sufficient to ensure equivalence of the input current or charge. For calibration purposes, it is generally necessary to establish that the same electrical quantity is applied to the reference instrument and instrument under test. Without such assurance, agreement in displayed values may not, by itself, demonstrate valid calibration.

Figure 1b illustrates a schematic of a charge source commonly used in Japanese standard laboratories. This configuration combines a microcurrent source (e.g., Keithley Model 6430) with a timer switch. The microcurrent source incorporates a servo circuit that maintains a constant voltage across the reference resistor, thereby enabling the generation of a stable current independent of the load resistance of the device under calibration. Additionally, the servo circuit includes a current‐limiting function that suppresses excessive output, helping to protect the electrometer from potential damage. Such servo mechanisms play an important role in ensuring both measurement stability and electrical safety.

In contrast, the current source proposed by Tsuno et al. does not appear to include an equivalent mechanism for regulating or limiting the output current. This difference may have implications for the stability of the generated current and the electrical safety of the measurement setup.

In light of these considerations, the suitability of the proposed current source for accurate and safe electrometer calibration may require further clarification. In related studies by Tsuno et al. published in Japan,^4^, ^5^ several commentaries have discussed similar technical considerations.^6^, ^7^, ^8^ Furthermore, the JSMP electrometer guidelines were subsequently revised to indicate that current sources whose output may vary depending on the electrometer under test should not be used for inspection or calibration.^2^

With respect to electrometer inspection and calibration, high‐performance microcurrent sources are commercially available and have been explored experimentally in Japan.^9^, ^10^ However, the number of published studies remains limited, and additional investigation would be valuable to further establish best practices in this area.

Sincerely yours,

## CONFLICT OF INTEREST STATEMENT

The author serves as the leader of the JSMP Electrometer Guidelines Working Group. The author declares no financial conflicts related to the subject of this manuscript.
